# Analysis of near infrared spectra for age-grading of wild populations of *Anopheles gambiae*

**DOI:** 10.1186/s13071-017-2501-1

**Published:** 2017-11-07

**Authors:** Benjamin J. Krajacich, Jacob I. Meyers, Haoues Alout, Roch K. Dabiré, Floyd E. Dowell, Brian D. Foy

**Affiliations:** 1 Arthropod-borne and Infectious Diseases Laboratory, Department of Microbiology, Immunology, and Pathology, Colorado State University, Fort Collins, CO USA; 2 Direction Régionale de l’Ouest (DRO), Institut de Recherche en Sciences de la Santé (IRSS), Bobo Dioulasso, Burkina Faso; 3 Stored Product Insect and Engineering Research Unit, United States Department of Agriculture/Agricultural Research Services, Center for Grain and Animal Health Research, Manhattan, KS USA

**Keywords:** *Anopheles*, Mosquitoes, Aging, Spectroscopy

## Abstract

**Background:**

Understanding the age-structure of mosquito populations, especially malaria vectors such as *Anopheles gambiae,* is important for assessing the risk of infectious mosquitoes, and how vector control interventions may impact this risk. The use of near-infrared spectroscopy (NIRS) for age-grading has been demonstrated previously on laboratory and semi-field mosquitoes, but to date has not been utilized on wild-caught mosquitoes whose age is externally validated via parity status or parasite infection stage. In this study, we developed regression and classification models using NIRS on datasets of wild *An. gambiae* (*s.l.*) reared from larvae collected from the field in Burkina Faso, and two laboratory strains. We compared the accuracy of these models for predicting the ages of wild-caught mosquitoes that had been scored for their parity status as well as for positivity for *Plasmodium* sporozoites.

**Results:**

Regression models utilizing variable selection increased predictive accuracy over the more common full-spectrum partial least squares (PLS) approach for cross-validation of the datasets, validation, and independent test sets. Models produced from datasets that included the greatest range of mosquito samples (i.e. different sampling locations and times) had the highest predictive accuracy on independent testing sets, though overall accuracy on these samples was low. For classification, we found that intramodel accuracy ranged between 73.5–97.0% for grouping of mosquitoes into “early” and “late” age classes, with the highest prediction accuracy found in laboratory colonized mosquitoes. However, this accuracy was decreased on test sets, with the highest classification of an independent set of wild-caught larvae reared to set ages being 69.6%.

**Conclusions:**

Variation in NIRS data, likely from dietary, genetic, and other factors limits the accuracy of this technique with wild-caught mosquitoes. Alternative algorithms may help improve prediction accuracy, but care should be taken to either maximize variety in models or minimize confounders.

**Electronic supplementary material:**

The online version of this article (10.1186/s13071-017-2501-1) contains supplementary material, which is available to authorized users.

## Background

Knowledge of the age structure of mosquitoes is critical to understand the spread of vector-borne disease. *Anopheles gambiae*, the major vector of malaria-causing *Plasmodium* spp. parasites, must undergo a 10–12 day extrinsic incubation period (EIP) during which a parasite develops into the human-infectious sporozoite stage and invades the salivary glands [1]. A large portion of the mosquito population is therefore unable to spread parasites, and very old mosquitoes are disproportionately important to the transmission cycle. Recent work has brought about the idea of “evolution-proof” insecticides that preferentially target older age classes of mosquitoes that have already exhausted most of their reproductive potential, but are at the peak of their disease-transmission potential [2]. Functionally, this approach can be performed through the use of existing insecticides in lower doses that would only be fatal to older, infection-stressed adults, or through fungal or biological control measures that shorten life or are disproportionately effective against older mosquitoes [3–6]. Rapidly assessing population-level age-structure is critical to evaluating the efficacy of these and other control endeavors, but there currently are limited tools available to do so [1].

Mosquito age-structure classifications have most often relied on female ovary dissections, especially characterizing the status of ovarian tracheoles [7, 8]. If a mosquito has not yet undergone a gonotrophic cycle (nulliparous), the tracheoles tend to be in tightly coiled “skeins.” However, if the tracheoles are unraveled, follicular development and oogenesis has likely occurred at least once (parous). This methodology has been utilized widely as it is a relatively simple dissection procedure, though it results in a coarse metric of age because many mosquitoes, particularly *An. gambiae,* become parous early in life. Thus, this method can only distinguish the very young from all other age classes. Further, this technique can be confounded by indeterminate ovaries due to an opaque residue after dissection, and ovaries that have a mix of coiled skeins and unraveled tracheoles on different ovarioles [9]. A subsequent dissection technique was developed by Polovodova [10, 11] that counts the ovarian dilations (relics of past egg batches) that can be found on the distal end of the ovariole. This technique is very technically demanding, requiring an injection of paraffin oil into the ovaries via the oviduct, and delicate removal of the ovary without damage [12]. Few researchers have successfully used this technique due to these limitations [13–15], and others have indicated that even when done successfully, the approach is flawed because of the presence of “rogue” ovarioles that indicate a gonotrophic cycle that did not occur [16, 17]. These non-diagnostic ovarioles increase in their frequency as the mosquito ages, and can also be confounded by taking multiple blood meals between age batches [18, 19]. All dissection approaches are also limited by the speed of the dissection, making high throughput processing difficult [20].

A range of alternative chemical and molecular approaches have been considered to address these limitations, including detection of fluorescent pteridines [21], changes in the ratio of cuticular hydrocarbons [22], transcriptomic variation [23], proteomic analysis [24], and recently the use of near-infrared spectroscopy (NIRS) [25]. NIRS is a fast and non-destructive technique that detects changes in the diffuse reflection of light within the near-infrared spectrum (780–2500 nm) due to the rotation, bending and stretching of C-H, N-H, O-H and other bonds [26]. This technique was first utilized for the study of moisture content of various grain species, but has recently been used with insects [26–29]. Mayagaya et al. [25] applied this approach to classify *An. gambiae* (*s.l.*) as young (< 7 days old) and old (≥ 7 days), and to identify them into *An. arabiensis* and *An. gambiae* (*s.s*.). Subsequently, this approach has been utilized with mosquitoes reared in semi-field enclosures and on some wild-caught adults, though importantly these wild-caught adults were not characterized by other methods (i.e. parity dissection or sporozoite analysis) [19, 30–32]. NIRS age-grading has demonstrated some robustness, with accuracy remaining consistent with varying developmental status (i.e. oviposition) [19]. However, species diversity, diet, physiological status, and rearing temperature may alter the accuracy of NIRS-based age grading techniques [19, 33–35]. These studies have found that the inclusion of a higher number of these variables in calibration models increases overall prediction accuracy when applied to varied test sets.

Lacking, to date, is an evaluation of NIRS’s age-grading ability with wild-caught vectors compared to classical measures of age-classification as external validators of age (parity status and the presence of sporozoites in the salivary gland). With a combination of these two independent measures, NIRS-predicted ages can be compared to known nulliparous (assumed young), parous (assumed mid-to-old), or sporozoite positive (known old) wild mosquitoes to validate and assess the accuracy of this methodology. In this study, we performed such an evaluation, using calibration datasets of *An. gambiae* (*s.l.*) generated from both laboratory colonies and wild larvae collected in the field to predict age classes with the above external validators.

## Methods

### Mosquito rearing

Two strains of laboratory-reared *An. gambiae* mosquitoes were utilized in this study: “CSU-IRSS” and “CSU-G3.” *An. gambiae* (*s.s*.) strain “CSU-IRSS” mosquitoes were recently colonized from field-caught larvae collected in southwestern Burkina Faso by the Institut de Recherche en Sciences de la Santé (IRSS), shipped to Colorado State University (CSU). *Anopheles gambiae* strain “CSU-G3” mosquitoes were originally colonized in 1975, and have been in colony at CSU for hundreds of generations [36]. Both colony strains were reared at 28 ± 2 °C and 80% humidity under a 14:10 light:dark photoperiod. Larvae were hatched in 15 l of tap water with ground Tetramin® fish food supplementation in 44-l bins.

Field-caught larvae were collected in the rainy season of 2013 and 2014 in natural pools in southwestern Burkina Faso around Soumousso (Latitude 11.01681, Longitude -4.052893), Kodeni (11.166667, -4.250000), Bougouriba (10.9313363, -3.6667348), and Diarkadougou (10.9014352, -3.5514027) with a mosquito dipper. Larvae and their collection water were placed in plastic water bottles and transferred to Bobo-Dioulasso, Burkina Faso (11.1727, -4.3304). They were placed into 44-l bins with water from the sources they were collected in for rearing, and kept outside under a shaded roof which exposed them to the natural variation of humidity and temperature present in the region.

Adult mosquito groups of roughly 100 per time point from both the laboratory and field were collected in 24 h emergence periods (day 0). They were separated via aspiration (InsectaZooka field aspirator - BioQuip Products, Rancho Dominguez, CA, USA), and placed in containers with a cotton ball soaked in 10% sucrose and water provided ad libitum. These mosquitoes were held for either 3, 6, 9, 12 or 15 days prior to scanning. Blood meals were offered via the arm of a human volunteer in compliance with the Helsinki Declaration (Colorado State University Institutional Review Board approval #09-1148H) at day 2 post-emergence, and the evening prior to their designated scan day (12–18 h later). Mosquito numbers of those successfully blood-fed in each calibration set are listed in Additional file 1: Table S1. Prior to scanning, all adult mosquitoes were classified under light microscopy to species by taxonomic key [37]. Samples kept for each dataset were based on collection amounts for each day. In general, sample sizes were held to 40 samples per time point in an effort to make the sample sizes equal across all days. In some instances this was impossible due to low sample numbers; in these cases all samples were held to a similar value (i.e. ~32 per time point in DS2).

### Near-infrared spectroscopy/scanning

At days 3, 6, 9, 12 and 15, mosquitoes were killed with triethylamine or chloroform before scanning (for all strains except CSU-G3). CSU-G3 were killed via freezing at -20 °C for approximately 30 min, and then left for another 30 min to equilibrate to room temperature (~25 °C). Our NIRS set-up and data processing largely follows previously published methodology [25]. Mosquitoes were placed on their dorsal side on a spectralon plate, and their head/thorax was scanned with a LabSpec4i spectrometer with a 3 mm bifurcated reflectance probe at a height of 3 mm (#ASD-135320-RevE - ASD Inc., Boulder, CO, USA). We centered the scan on the head/thorax to limit the effects of the blood meal itself on the spectra, though note there are significant changes to protein expression and other factors with blood-feeding [38]. The software was set to take 20 spectra from each mosquito which it stores as an average spectrum. Absorbance values are recorded from 350 to 2500 nm. All scanning was performed within 6 h of the end of collection, with mosquitoes being kept alive until immediately prior to knock down/scanning. Delays between collection times and scanning are due to travel time, and should have no differences between groups. All field samples were scanned indoors in Bobo Dioulasso, Burkina Faso, and all laboratory samples were scanned indoors at Colorado State University. No preservation approaches were utilized as mosquitoes were scanned shortly after collection and immediately after they were killed.

### Data analysis and model creation

Spectra were converted to text using ViewSpec Pro version 6.2 (ASD Inc.) as wavelength *vs* Log(1/R). Spectra were manually viewed using the IQ Predict software, and any spectral profiles that lacked distinct absorbance peaks due to poor positioning or poor quality of the specimen were discarded from analysis (0–3.9% of samples depending on dataset). Six different sample sets were created (Additional file 1: Table S1) to represent a range of collection locations and groupings. These included two datasets that were combinations of field mosquito datasets (DS5) and field mosquito datasets plus the recently colonized strain dataset (DS6). The CSU-G3 dataset (DS4) was left out of these mixed datasets due to the difference in knockdown technique (freezing *vs* chemical anesthesia). Only the region from 500 to 2350 nm was utilized in analyses to remove regions of poor sensor sensitivity, and all spectra were pre-processed via mean centering using the ‘*caret*’ package in R version 3.3.2 using the RStudio 1.0.44 [39–41].

A range of regression (providing numeric values) and classification (grouping into a descriptive class of age of ‘young’ or ‘old’) algorithms were utilized for sample analyses (see Table 1 for a list of all algorithms used). Additionally, we included the use of full-spectrum PLS as it has been most commonly utilized for the age-grading of insect species. All algorithms are assessed for accuracy using the root mean squared error metric (RMSE), which allows for assessment of overall predictive accuracy in a value with interpretable units (i.e. “days”). The partial least squares (PLS) [42], support vector machine using a linear kernel (svmLinear) [43], and oblique random forest (ObliqueRF) [44] algorithms were implemented using the ‘*caret*’ package. The number of latent variables (nLV) used in PLS models was chosen based upon the lowest root mean squared error of 5-fold cross-validation with a maximum of 10 latent variables. The parameters for svmLinear and ObliqueRF were tuned using the adaptive resampling search in ‘*caret*’. The interval PLS (iPLS) [45, 46] algorithm was implemented using the ‘*mdatools*’ package [47]. This method uses intervals of 60 nm across the full spectra (500–2350 nm), maximizing accuracy while keeping the fewest intervals possible based on Wold’s R criterion. The ensemble PLS (enPLS) algorithm with variable selection was performed using the ‘*enpls*’ package in R [48]. In enPLS, the most informative wavelengths were chosen via Monte-Carlo uninformative variable elimination as in Cai et al. [46, 49, 50] (Additional file 2: Figure S1). Additionally, two methodologies were analyzed that use the model population analysis framework for chemometrics [51], in which sub-regression models are created to analyze the importance of variables (wavelengths) and samples (to evaluate outliers).

**Table 1:** Algorithms used in analysis

Algorithm	Used for regression or classification?	Outlier detection?	Variable selection?
Partial Least Squares (PLS)	Both	No	No
interval PLS (iPLS)	Regression	No	Yes
ensemble PLS with feature selection (enPLS)	Regression	Yes	Yes
Model Adaptive Space Shrinkage - PLS (MASS)	Regression	Yes	Yes
Variable Combination Population Analysis (VCPA)	Regression	No	Yes
Support Vector Machine-Linear Kernel (svmLinear)	Both	No	No
Oblique Random Forest - Ridge (ORF)	Classification	No	No

The first, Model Adaptive Space Shrinkage-PLS (MASS) provides simultaneous variable selection and outlier detection, and was implemented in MATLAB R2015a (The MathWorks, Inc., Natick, Massachusetts, USA) using author-provided code with 10-fold cross-validation [52]. Variable Combination Population Analysis (VCPA) which provides more aggressive variable selection, was also implemented in MATLAB with author provided code and 5-fold cross-validation [53]. The results of the root mean squared error of calibration (RMSEC, the self-prediction of the full dataset), the root mean squared error of cross-validation (RMSECV, the average prediction of 5 or 10-fold cross-validation where 80 or 90% of the model predicts the 20 or 10% left out that allows for parameter tuning to improve prediction), the root mean squared error of validation (RMSEV, the error of prediction of the 20% of the spectra left out prior to model creation that the parameters are not tuned on), and the root mean squared error of prediction (RMSEP, the error of a fully independent test set collected on different days) are presented for all the regression models presented.

Finally, we included three classification-based models (classifying mosquito ages of 3 and 6 days as “young” and days 9, 12 and 15 as “old”). This was done to recapitulate the < 7 or > 7 day grouping method used in other mosquito-age prediction NIRS literature [25]. These algorithms were chosen to compare the more commonly utilized algorithm (PLS), an alternative linear classifier (svmLinear), and a tree-based classifier reported to have success with spectral data (ObliqueRF) [54].

All R and MATLAB code and unprocessed spectral files are available for download at the link provided in the “Availability of data and materials” section below.

### Validation and independent test sets

Validation sets (Additional file 3: Table S2) were created by choosing ~20% of the spectra to leave out of the training set prior to model creation using the “Pick me!” random file selection software (Matías Nahuel Carballo) [55]. As above, we attempted to hold sample sizes consistent across days. Means for each day were calculated and analyzed via an analysis of variance (ANOVA) test with Tukey’s multiple comparisons adjustment with GraphPad Prism v6.1 (GraphPad Software, San Diego, CA). Classification models were tested using Acc > NIR metric of McNemar’s Chi-square test in the ‘*caret*’ package.

Independent test sets (Additional file 4: Table S3) were utilized to determine overall prediction accuracy on a non-biased sample set [25]. These samples were considered to be independent as they were reared separately, and were from unique collection days and/or locations relative to samples in the calibration dataset [35, 56]. This independence distinguishes them from validation sets. Independent test set 1 (ITS1) was comprised of adults reared with access to sugar and human blood meals after being collected from various larval sources from Burkina Faso in 2013 and 2014 and held for the recorded amount of time post-eclosion; thus the exact ages are known. Due to limited collection numbers and survivorship, the day 15 group is not included in ITS1.

Independent test sets 2 and 3 (ITS2, ITS3) were comprised of adult mosquitoes caught via indoor aspiration or by a human baited tent-trap and were noted for blood-fed status, scanned, parity dissected (see below), and stored in individual 1.5-ml tubes containing t.h.e. 100% indicating silica gel desiccant beads (#EM-DX0017–1, EMD Millipore, Billerica, MA, USA) for sporozoite analysis (see below) [57, 58]. ITS3 consisted of the nulliparous compared to the parous mosquitoes, while ITS2 consisted of the nulliparous mosquitoes compared to the sporozoite positive mosquitoes. Accuracy for classification algorithms for ITS1–3 is based on whether or not the model successfully classified mosquitoes < 7 days old or that are nulliparous as “early” or mosquitoes > 7 days old, parous, or sporozoite positive as “late.” This classification is not based on physiological status, but rather the age or predicted age of these groups.

To test the role blood-fed status has on model prediction, an additional split of DS6 was performed into non-blood-fed and blood-fed mosquitoes, comparing these models to only members of TS1-TS3 that match their blood-meal status.

### Parity dissection and sporozoite analysis

For an externally validated test set, groups of approximately 20 wild adult mosquitoes per day were caught via aspiration in the villages of Bougouriba and Diarkadougou, Burkina Faso in 2013 and were dissected to assess parity status via Detinova’s method under light microscopy [8, 59]. A random selection of these samples was then created based on the number of samples collected for each class (Additional file 4: Table S3). From the saved head/thorax, DNA was extracted with the 96-well format DNeasy Blood and Tissue Kit (#69504, Qiagen, Hilden, Germany), and analyzed for the presence of *Plasmodium* spp. sporozoites via Taqman quantitative-Polymerase Chain Reaction (qPCR) [60].

## Results

### Feature/variable selection and outlier detection

**Fig. 1:**
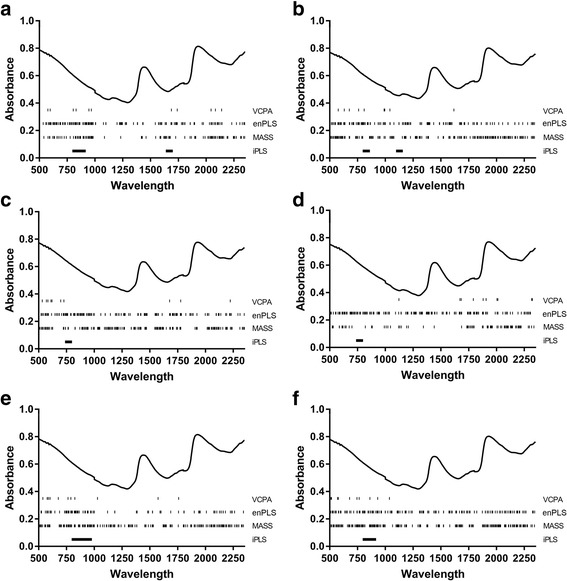
Averaged spectra per dataset, and wavelengths utilized by variable selection approaches. Datasets 1–6 are displayed in panels **a–f**, respectively. Wavelengths selected by the four algorithms are represented by the tick marks under the spectral profile

Four of the methods investigated reduced the variable space from the maximum 1851 spectral wavelengths (i.e. 500–2350 nm) via a range of approaches. VCPA had the most aggressive selection approach, reducing the variable space to 10–12 wavelengths. MASS tended to keep the largest number of variables of these selection methods (140–482 wavelengths) (Table 2). In general, the region from 500 to 1000 nm was represented most heavily in the majority of datasets, though few wavelengths or sets of wavelengths were consistent between all algorithms or datasets (Fig. 1). However, while the iPLS predictions for datasets 3 and 4 chose the same interval as having the lowest RMSE value in cross-validation (740–799 nm), there was no ability of these models to predict each other (data not shown). In general, the prediction accuracy of the feature reduction methodologies was higher than that of PLS, though not necessarily other full-spectra prediction algorithms such as svmLinear (Table 2).

**Table 2:** Calibration, cross-validation, validation and independent test set 1 (ITS1) results for each algorithm on the 6 datasets

Dataset	Samples	No. var	RMSEC	R^2^Cal	RMSECV	R^2^CV	LV	RMSEV	RMSEP-ITS1
Dataset 1									
PLS	178	1851	2.68	0.55	3.16	0.39	10	2.90	3.88
iPLS	178	180	2.41	0.64	2.92	0.55	10	2.97	5.52
enPLS	175	400	1.71	0.82	2.04	0.74	na	2.62	7.01
MASS	173	258	2.00	0.74	2.28	0.66	10	2.93	4.04
VCPA	178	11	2.36	0.65	2.52	0.60	10	3.11	4.64
svmLinear	178	1851	na	na	2.83	0.59	na	2.70	4.29
Dataset 2									
PLS	156	1851	1.85	0.83	2.28	0.74	10	2.71	4.08
iPLS	156	120	1.54	0.93	1.20	0.90	10	2.41	3.88
enPLS	152	300	0.81	0.97	1.05	0.95	na	1.89	4.19
MASS	153	385	0.87	0.96	1.10	0.94	10	2.41	4.33
VCPA	156	10	1.88	0.82	2.08	0.78	10	2.49	3.29
svmLinear	156	1851	na	na	1.89	0.81	na	2.13	4.60
Dataset 3									
PLS	160	1851	2.05	0.80	2.61	0.70	10	2.85	5.53
iPLS	160	60	1.97	0.81	2.41	0.78	10	2.29	5.61
enPLS	158	350	0.76	0.97	1.44	0.90	na	1.96	4.29
MASS	158	441	1.24	0.93	1.59	0.88	10	2.06	4.17
VCPA	160	10	1.95	0.82	2.05	0.80	8	2.55	3.40
svmLinear	160	1851	na	na	1.94	0.82	na	2.23	3.76
Dataset 4									
PLS	200	1851	2.10	0.76	2.60	0.64	10	2.43	5.18
iPLS	200	60	1.71	0.84	2.17	0.80	10	2.41	4.05
enPLS	195	350	0.85	0.96	1.32	0.90	na	1.49	3.56
MASS	196	140	1.55	0.87	1.78	0.82	10	1.98	3.95
VCPA	200	11	2.28	0.71	2.39	0.69	7	2.72	6.44
svmLinear	200	1851	na	na	1.99	0.77	na	1.74	4.32
Dataset 5									
PLS	334	1851	2.94	0.50	3.16	0.43	10	3.42	3.57
iPLS	334	180	2.50	0.64	2.76	0.58	10	2.72	6.70
enPLS	330	200	1.77	0.82	2.07	0.75	na	3.10	4.69
MASS	329	466	2.20	0.71	2.36	0.67	10	3.10	3.67
VCPA	334	12	2.82	0.54	2.89	0.51	8	3.70	4.79
svmLinear	334	1851	na	na	2.66	0.63	na	2.81	3.70
Dataset 6									
PLS	494	1851	3.24	0.43	3.50	0.34	10	3.29	3.43
iPLS	494	120	3.21	0.44	3.36	0.41	8	2.99	5.01
enPLS	479	300	1.76	0.83	2.21	0.73	na	2.77	3.33
MASS	492	482	2.58	0.64	2.83	0.56	10	3.08	2.96
VCPA	494	10	3.43	0.47	3.15	0.46	10	3.43	2.48
svmLinear	494	1851	na	na	2.68	0.61	na	2.78	3.49

*Note*: eEach of the six datasets were used to generate models using six regression algorithms. The root mean squared error (RMSE) is presented for the calibration, cross-validation and validation sets, and independent test set 1. This measure (with units of “days”) allows for an approximation of how much error is present across the range of ages present in each dataset

*Abbreviations*: *No. of var.* number of variables used, *RMSEC* root mean squared error of calibration, *R* ^*2*^ *Cal* coefficient of variation of calibration, *RMSECV* root mean squared error of cross-validation, *R* ^*2*^ *CV* coefficient of variation of cross-validation based on the actual *vs* predicted ages of the average of the 5 or 10 fold cross-validation, *LV* number of latent variables used in PLS regression (if applicable), *RMSEV* root mean squared error of validation set, *RMSEP-ITS1* root mean squared error of prediction for independent test set 1, *na* not available for RMSEC/ R^2^Cal values (was not calculated natively in the implementation of svmLinear) or not applicable for LV (due to use of ensemble models in enPLS and not used in support vector machines)

Outlier detection was available in two of the six methodologies investigated. Few outliers were marked by either method, with a maximum of 3.03% removed in dataset 6 with enPLS (Table 2). Methodologies with outlier detection did not appear to have an obvious increase in prediction accuracy over other methods with our datasets.

### Accuracy of calibration, cross-validation, and validation set prediction

**Fig. 2:**
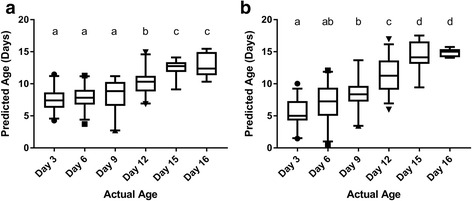
Predicted *vs* actual age for NIRS validation set 6 (VS6) with two models. Partial least squares (**a**) and ensemble partial least squares (**b**) are displayed. 25–75% confidence (box) and 5–95% confidence intervals (whiskers) are marked. Groups with statistically different means (*P* < 0.05) via ANOVA with Tukey’s multiple comparisons adjustment are marked with different letters

Calibration sets allow for an initial assessment of model fit, and for a value to compare cross-validation accuracy to have an indication of overfitting. If not generated natively via the algorithm, this value was not presented (svmLinear). Of the other five algorithms, enPLS had the lowest RMSEC for each of the 6 datasets, followed by MASS. In cross-validations, the root mean squared error varied from 1.05–3.50 days depending on the dataset and algorithm used (Table 2). Additionally, the R^2^ value, calculated based on the actual *vs* predicted values for each of the ages, followed the RMSE (i.e. lower RMSE has higher R^2^). The highest errors were predominantly found in the multi-source datasets (DS5 and DS6), likely due to the increase in genetic variation and sampling locations included in these sets. Of the six algorithms investigated, PLS was the least accurate, having the highest root mean squared errors among algorithms in the cross-validation testing set (Table 2). The most accurate algorithm for cross-validation sets was enPLS for 5 of 6 sets tested. The root mean squared error of the validation set was lowest for the enPLS model for 3 of 6 datasets. In general, the validation sets tended to have over-predicted ages of younger mosquitoes (+4.54 and +1.93 days for day 3 and day 6 with PLS dataset 6, respectively) (Fig. 2), though this difference was reduced by the ensemble PLS algorithm (+2.50 and +1.00 days). Older mosquitoes tended to be slightly under-predicted (-1.78 days for day 12, -2.62 for day 15, -3.00 for day 16 with PLS; -0.65 for day 12, -0.67 for day 15, and -1.08 for day 16 with SVM). These under/over-prediction trends follow what has been reported previously with age-classification of insects with NIRS [25, 33, 34]. Classification algorithms accurately grouped validation sets at a minimum of 70.7% to a maximum of 98.0% correctness (Table 3). For dataset 6, accuracy was highest with Oblique Random Forest and svmLinear (85.4% and 83.7% classified correctly, respectively).

**Table 3:** Classification model accuracy for cross-validation, validation, and independent test sets. The classification accuracy, i.e. was a mosquito whose actual age was less than 7 days of age or greater than 7 days of age predicted as “young” or “old,” respectively in cross-validation, validation, or ITS1; or the accuracy of predicting a nulliparous mosquito successfully as “young”, a parous mosquito as “old”, or a sporozoite positive mosquito as “old” (ITS2 and ITS3) is presented. All classifications within sets are binary (i.e. young *vs* old). If accuracy was significant via McNemar’s Chi-square test, the 5–95% confidence interval is presented in the parenthesis. Degree of significance is demarcated

Dataset	Accuracy CV	Accuracy V	ITS1 Accuracy	ITS2 Accuracy	ITS3 Accuracy
Dataset 1					
PLS	0.7913	0.7727 (0.6216–0.8853)**	0.5507	0.5625	0.5128
ObliqueRF	0.8649	0.7955 (0.647–0.902)***	0.5652	0.625 (0.5096–0.7308)*	0.5128
svmLinear	0.8422	0.8636 (0.7265–0.9483)***	0.6232 (0.4983–0.7371)*	0.6232 (0.4983–0.7371) *	0.5128
Dataset 2					
PLS	0.9165	0.8421 (0.6875–0.9398)***	0.4493	0.6 (0.4844–0.708)*	0.5385
ObliqueRF	0.9354	0.8684 (0.7191–0.9559)***	0.4058	0.55	0.5385
svmLinear	0.9356	0.8947 (0.752–0.9706)***	0.4348	0.6 (0.4844–0.708)*	0.5769
Dataset 3					
PLS	0.95	0.878 (0.738–0.9592)***	0.5072	0.4625	0.4872
ObliqueRF	0.9687	0.9756 (0.8714–0.9994)***	0.5942	0.55	0.4744
svmLinear	0.9562	0.9756 (0.8714–0.9994)***	0.5217	0.5375	0.4872
Dataset 4					
PLS	0.895	0.88 (0.7569–0.9547)***	0.4928	0.5	0.5128
ObliqueRF	0.97	0.98 (0.8935–0.9995)***	0.5072	0.525	0.4615
svmLinear	0.945	0.96 (0.8629–0.9951)***	0.5362	0.55	0.4744
Dataset 5					
PLS	0.7726	0.7073 (0.5965–0.8026)***	0.5942	0.55	0.5385
ObliqueRF	0.8442	0.7805 (0.6754–0.8644)***	0.6667 (0.5429–0.7756)**	0.525	0.4872
svmLinear	0.8232	0.8049 (0.7026–0.8842)***	0.6812 (0.5579–0.7883)**	0.5875	0.5769
Dataset 6					
PLS	0.7348	0.748 (0.6617–0.8219)***	0.6812 (0.5579–0.7883)**	0.55	0.4872
ObliqueRF	0.8502	0.8537 (0.7786–0.9109)***	0.6232 (0.4983–0.7371)*	0.625 (0.5096–0.7308) *	0.5256
svmLinear	0.8518	0.8374 (0.7601–0.8978)***	0.6957 (0.5731–0.8008)**	0.5625	0.5

*P < 0.05, **P < 0.01, ***P < 0.001

*Abbreviations*: *CV* cross-validation, *V* validation, *ITS* independent test set, *LV* latent variables used if applicable

### Accuracy on independent test set 1

**Fig. 3:**
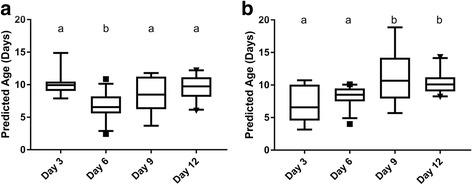
Comparison of predicted *vs* actual age for independent test set 1 (ITS1) with two models. Partial least squares (**a**) and ensemble partial least squares (**b**) are displayed

Independent test set 1 consisted of 69 mosquitoes collected as wild-caught larvae, with 57 of 69 being fully or partially blood-fed. Accuracy for the three independent test sets varied considerably based on which set and model was used. The lowest root mean squared error of prediction for independent test set 1 was with the VCPA model built on dataset 6 (Table 2). This level of error allowed for discrimination between young and old mosquito ages (3 and 6 compared to 9 and 12 days post-emergence), though this difference was only significant between Days 3 to 12, 6 to 9, and 6 to 12 via Tukey’s multiple comparisons test. This was predominantly due to an over-prediction of Day 3 mosquitoes (+4.15 days) and an under-prediction of Day 12 mosquitoes (-2.43 days). The regression model with the clearest delineation between young and old mosquitoes from the independent test set was the ensemble PLS model created from dataset 6 (Fig. 3b). With classification algorithms for independent test set 1, the svmLinear model had the best accuracy (Table 3). Misclassification (being classed as “old” for days 3 and 6 or “young” for days 9 and 12) was spread relatively evenly across days (37.5% misclassified Day 3, 29.6% Day 6, 15.4% Day 9, and 38.1% Day 12).

### Accuracy on externally validated independent test sets (independent test sets 2 and 3)

**Fig. 4:**
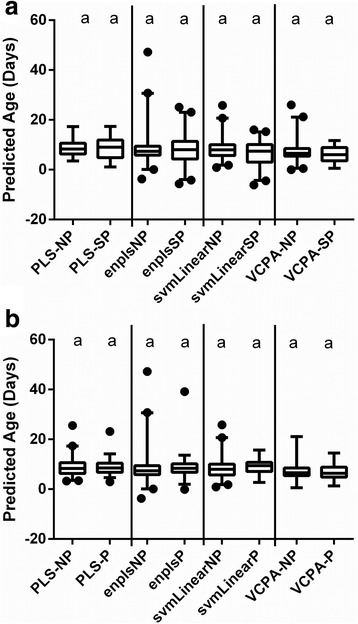
Prediction of independent test set 2 (ITS2) (nulliparous *vs* *Plasmodium* sporozoite positive, **a**) and independent test set 3 (ITS3) (nulliparous *vs* parous, **b**) for five algorithms created from dataset 6

In total, 40 nulliparous, 39 parous, and 40 sporozoite positive (all *P. falciparum*) *An. gambiae* were used for independent test sets. These numbers were chosen to roughly keep classes the same size, with the sporozoite positive samples being the limiting sample set (due to a 1.5–5.1% sporozoite positive rate in samples tested) [58]. Of these, 28/40 of the nulliparous are non-blood-fed, 27/39 of parous are non-blood-fed, and 8/40 of the sporozoite positive are non-blood-fed (Additional file 5: Table S4). As these are field mosquitoes caught as adults, the exact ages of these samples are unknown. Due to this, we are unable to calculate the RMSEP, and instead compare mean age predictions for each class (i.e. nulliparous, parous, or sporozoite positive) using the regression algorithms with the highest prediction accuracies for independent test set 1 (all dataset 6 models except iPLS due to poor predictive power on ITS2) (Table 2). None of the regression models used predicted a difference in means between nulliparous and sporozoite positive mosquitoes via Sidak’s multiple comparisons test (Fig. 4a), or for nulliparous and parous mosquitoes (Fig. 4b). Only a few algorithms were able to discriminate nulliparous from sporozoite positive mosquitoes at a statistically significant level via McNemar’s Chi-square test (*P* < 0.05), though the maximum accuracy was only 62.5% with ObliqueRF from DS1 and DS6.

## Discussion

In this study we assessed the use of NIRS for the age-grading of wild-caught mosquitoes whose age had been externally validated by existing approaches. Accuracy for cross-validation and validation datasets indicated that there may be some loss in accuracy due to high complexity present in the datasets (i.e. the highest RMSECV and RMSEV values were found in datasets 5 and 6). This slight decrease in self-predictive accuracy would be expected as models trained on a diverse set of data should not capture all of the diversity present, otherwise they would be over fit to the data [61]. The best measure of success then should come through independent test sets that are not generated from calibrated data (i.e. not the 20% left out for validation or the 5/10-fold cross-validation sets used for model parameter tuning). Unfortunately, we found difficulty in accurate prediction of most of the independent test sets including wild-caught mosquitoes reared to known ages (independent test set 1, ITS1), and mosquitoes whose age had been externally validated by parity or presence of sporozoites (independent test sets 2 and 3, ITS2 and 3). Using the full-spectrum PLS algorithm, there was limited predictive power with any of the six datasets for ITS1, ITS2 or ITS3, except for PLS-classification on ITS1. Alternative algorithms such as VCPA and MASS had some improvement on these test sets for regression-based prediction, largely on independent test set 1 (Table 2), however overall accuracy was low with all algorithms. The best delineation between points came with the largest dataset (dataset 6) discussed further below. With these regression models however, we had no indication of successful discrimination of independent test set 2 (nulliparous *vs* sporozoite-positive) or independent test set 3 (nulliparous *vs* parous). There was some success with the classification models with accuracy of cross-validation and validation being comparable with the de facto classification (< 7, ≥ 7 day) age prediction used previously [25], although this accuracy was still relatively low (Table 3).

Several physical or life history reasons may contribute to the low predictive power we observed. As mentioned above, it has been found that diet and physiological status can impact the accuracy of age prediction on insects, and that inclusion of a greater number variety of samples in the calibration model may improve accuracy [19, 34, 35]. It was then the hope that the datasets presented here that spanned a range of years, capture locations, and larval conditions, while keeping the instrument parameters stable, would allow for the creation of a robust model with broad predictive power. We did attempt to time blood-feeds to be roughly 8–12 h before the scan, as this would be analogous to the primarily blood-fed wild-caught vectors collected via aspiration. A caveat to sampling is that we did include some partially blood-fed or non-blood-fed mosquitoes in models to increase numbers for sporozoite detection as this is a low prevalence population, and also for ease in parity dissection. While it is possible this somewhat limits predictive power, controlling for blood-fed status did not increase predictive power (Additional file 6: Table S5; Additional file 7: Figure S2).

Additionally, use of natural water sources for mosquito rearing (datasets 1 and 2) was used to limit the confounding factor of larval diet and geographical factors for prediction. As models built on dataset 6 had the highest test set prediction accuracy, accounting for these factors may be important. Future models could also include very young (day 1) mosquitoes, though as it was unlikely that these recently emerged mosquitoes would be collected with the trapping methods used in this study, they were not included [62].

Other possible confounders limiting predictive power may be due to the external validators themselves. It is possible that through a late first blood meal or heterogeneity in gonotrophic development [63], some mosquitoes may be old but nulliparous. Additionally, if mosquitoes very rapidly feed post-emergence and have already been inseminated which has been shown to be common in nulliparous mosquitoes [64], they may become parous early in life causing overlap with nulliparous mosquitoes. As an additional test, we tried the classification models with an alternative split of “early” and “late” samples, including six day-old mosquitoes as “late” as the mean age of nulliparous mosquitoes has been previously reported to be 3–4 days old [65]. This largely did not improve accuracy in classification (Additional file 8: Table S6), however may have provided a slight increase in predictive ability for svmLinear on ITS2 and ITS3. Also, it has been found that through inadvertent inclusion of midgut tissue infected with *Plasmodium*, a mosquito could be called as sporozoite positive [66]. However, we had known of this study and were careful when splitting the head and thorax from abdomen to avoid this.

Finally, there is possibility that, the *Plasmodium* infection itself, or the gonotrophic process/physiology could alter the spectroscopy signal [19]. The calibration mosquitoes were kept with males, so mating could occur though oviposition papers were not provided. As the calibration mosquitoes were fed on uninfected human volunteers there would be no *Plasmodium* infection in these mosquitoes, and so this possible confounder is unaccounted for in the models. Likely many of these factors are small in their impact on the overall evaluation of the methodology, however their presence cannot be fully discounted and future studies should attempt to control for them where possible. Some evidence indicates that there is something innate about the samples causing them to be underpredicted, as all methodologies for age discrimination correlate strongly (*P* < 0.0001, Pearson’s r) (Additional file 9: Figure S3).

Another possible methodological cause for the difficulty in this cross-population prediction may be seen in Fig. 1, which shows that there was limited overlap between feature/variable selection algorithms with and between datasets. There was some clustering in the region from 500 to 1000 nm, though this was not found for every algorithm and dataset (Fig. 1, VCPA, enPLS and iPLS algorithms). This differs somewhat from what was presented previously in Mayagaya et al. [25] based on PLS regression coefficients that found a more broad distribution of importance of wavelengths “700, 1000, 1221, 1305, 1412, 1728, 1878, 1947 and 2200 nm”. Additionally, the poor predictive ability may not solely be based on wavelength selection via the algorithms, as full spectrum models and models that selected the same wavelengths (i.e. iPLS for datasets 3 and 4) had poor ability to predict other datasets (data not shown).

We attempted in a range of ways to cope with these and other issues in our analytical methodology. The first is that through using methods that provide variable selection we could help to address the “small n, large p” problem in which we have relatively few sample numbers but many predictors which can easily lead to overfitting of models [67]. However, while the variable selection methods had more accuracy than PLS alone, overall they were unable to create a parsimonious model that had robust and broad predictive success. Due to this failure, we would suggest in future experiments that dataset complexity be reduced prior to model creation as much as possible. As it has been previously reported [25], members of *An. arabiensis* and *An. gambiae* (*s.l.*) can be delineated via NIRS. Mosquitoes from 2013’s calibration model (DS1) were able to be identified, with 68.0% found to be *An. arabiensis* (data not shown). Due to insufficient sample numbers in these periods, we were unable to split these data into two calibration models for each identified species. Additionally, attempts to create a predictive model to distinguish *Anopheles* species via NIRS, as in the Mayagaya et al. [25] paper, were unsuccessful with our samples that had been identified to species (data not shown). Unfortunately, we were unable to reliably classify mosquitoes as *An. arabiensis* or *An. gambiae* (*s.s*.) by PCR from the 2014 collection year in Burkina Faso likely due to post-NIRS degradation of the samples, as they were stored dried in tubes with desiccant and kept at room temperature [68]. The ability to classify groups of mosquitoes based on species may have improved our accuracy as each of the models could have been specific to one species. With this in mind, future studies could be improved by accounting for this variation, and the recent species delineation of *An. gambiae* (*s.s*.) and *An. coluzzii* [69]. To our knowledge, there has been no publication demonstrating the ability to distinguish *An. gambiae* (*s.s*.) and *An. coluzzii* with near-infrared spectroscopy, though this would possibly allow for the avoidance of more laborious PCR-based identification. Additional measures that could be added are data pre-processing methods that may improve predictive performance [70]. Unfortunately there is no easy way to simplify determination of which combinations of preprocessing and algorithms will provide best results for datasets as has been popularized by the phrase there is “no free lunch” to model selection [71]. Finally, while the same machine was utilized for all scanning performed, the samples were scanned “on location” and thus there may be some temperature or other differences year-to-year and day-to-day between groups which could impact results [34].

## Conclusions

The utilization of NIRS for age-grading of wild mosquitoes is a complex problem that remains unsolved by our extensive analyses presented here. Increasing of sample diversity, new prediction algorithms, and a reduction of other confounders may improve outcomes, though in this study the reliability of this approach was insufficient for broader use. We suggest a greater control of physiological status, use of local larvae, and careful determination of species in the calibration steps to improve the accuracy of the technique. Most importantly, however, this study shows the importance of having external validators for evaluation of success in calibration models. Without some form of external validation, the values generated by NIRS may be questionable due to the variation present in wild samples.

## Additional files

Additional file 1: Table S1. Dataset numbers. Calibration Model sample size and collection locations of single (DS1-DS4), and multi-source (DS5-DS6) origin. Soumousso and Kodeni are located in southwestern Burkina Faso. (XLSX 10 kb)

Additional file 2: Figure S1. Example figure of selection of optimal number of variables for ensemble PLS. The lowest root mean squared error of cross-validation for the fewest number of variables was used for prediction of test sets. In this example, 300 variables was chosen, as it has lower error in cross-validation compared to other variable amounts (including the full spectra - 1851 variables). (TIFF 681 kb)

Additional file 3: Table S2. Validation Model sample size and collection locations. (XLSX 10 kb)

Additional file 4: Table S3. Independent test set sample size and collection locations. *Abbreviations*: DK, Diarkadougou, Burkina Faso, “Unknown” was from a collection in southwestern Burkina Faso; BG, Bougouriba, Burkina Faso; NP, nulliparous via dissection; P, parous; SP, *Plasmodium* sporozoite positive via qPCR. (XLSX 9 kb)

Additional file 5: Table S4. Full listing of ITS1–3 Blood-fed status, metadata, and spectral data. Each ITS is listed on a new tab of the .xlsx file. *Abbreviations*: BF, blood-fed; PBF, partially blood-fed; NBF, non-blood-fed; Loc, location and trapping method; Sporo: sporozoite (nulliparous with value of 1, positive with 2; for ITS3 nulliparous mosquitoes have a value of 1, parous value of 2). (XLSX 5810 kb)

Additional file 6: Table S5. Blood-fed status on regression accuracy for ITS1. Root mean squared error of prediction for ITS1 with three algorithms. (XLSX 8 kb)

Additional file 7: Figure S2. Predicted ages of same blood-meal status (i.e blood-fed or non-blood-fed) mosquitoes from ITS2 using models generated from only blood-fed or only non-blood-fed calibration mosquitoes from DS6. *Abbreviations*: BF, blood-fed; NBF, non-blood-fed; SVM, SVMLinear; PLS, partial least squares; iPLS, interval PLS. (TIFF 92 kb)

Additional file 8: Table S6. Classification model accuracy with 6-day old mosquitoes classified as “late”. The original and adjusted tables for dataset 6 are presented. *P*-values for the alternative classification table are listed. Degree of significance is demarcated (*P < 0.05, **P < 0.01, ***P < 0.001). *Abbreviations*: CV, cross-validation; V, validation; ITS, independent test set; LV, latent variables used if applicable. (XLSX 10 kb)

Additional file 9: Figure S3. Correlation plot of ages of sporozoite-positive mosquitoes. The predicted ages of expected old, sporozoite-positive mosquitoes for each of the four algorithms are shown. Partial least squares compared to enpls, svmLinear, VCPA (**a**), enpls compared to svmLinear and VCPA (**b**), and svmLinear compared to VCPA **(c)** are shown. All models correlated at *P <* 0.0001 via Pearson’s r. (TIFF 434 kb)

## Abbreviations

ANOVA: Analysis of variance
CSU: Colorado State University
DS1-DS6: Datasets 1–6
EIP: Extrinsic incubation period
enPLS: Ensemble PLS
iPLS: Interval PLS
IRSS: Institute de Recherche en Sciences de la Santé
ITS1-ITS3: Independent test sets 1–3
MASS: Model adaptive space shrinkage
MDA: Mass drug administration
NIRS: Near infrared spectroscopy
ORF: Oblique random forest
PLS: Partial least squares
PRESS: Predicted residual sum of squares
qPCR: Quantitative-polymerase chain reaction
RMSEC: Root mean squared error of calibration
RMSECV: Root mean squared error of cross-validation
RMSEP: Root mean squared error of prediction
RMSEV: Root mean squared error of validation
SECV: Standard error of cross-validation
SVM: Support vector machine
VCPA: Variable combination population analysis
