# Supplementary figures and images for: Analysis of near infrared spectra for age-grading of wild populations of Anopheles gambiae
Source: Parasit Vectors. 2017 Nov 7;10:552. doi: 10.1186/s13071-017-2501-1 (PMC5678599; doi:10.1186/s13071-017-2501-1)

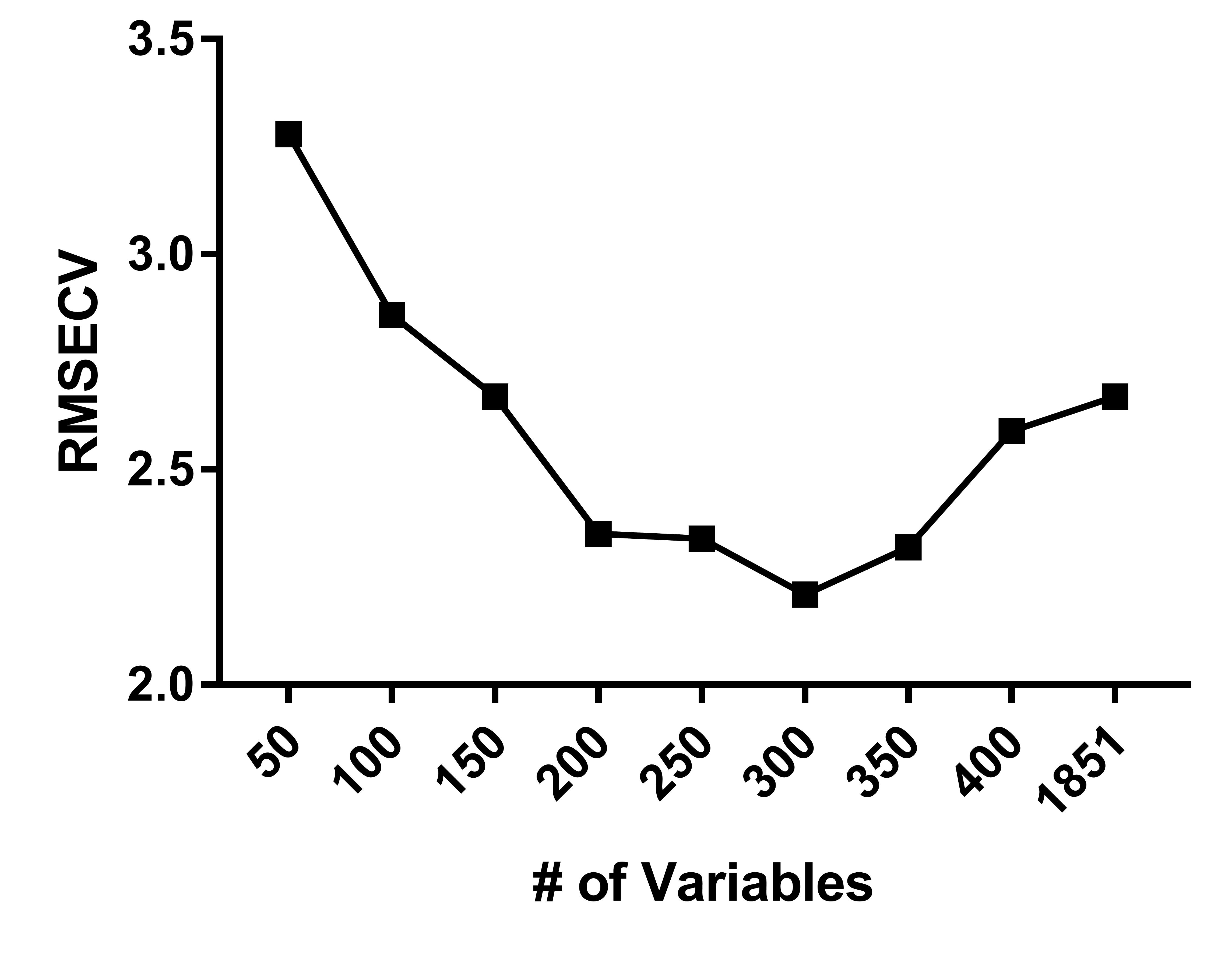

Supplement: Supplementary file 2 — Example figure of selection of optimal number of variables for ensemble PLS. The lowest root mean squared error of cross-validation for the fewest number of variables was used for prediction of test sets. In this example, 300 variables was chosen, as it has lower error in cross-validation compared to other variable amounts (including the full spectra - 1851 variables). (TIFF 681 kb) [file 13071_2017_2501_MOESM2_ESM.tif]

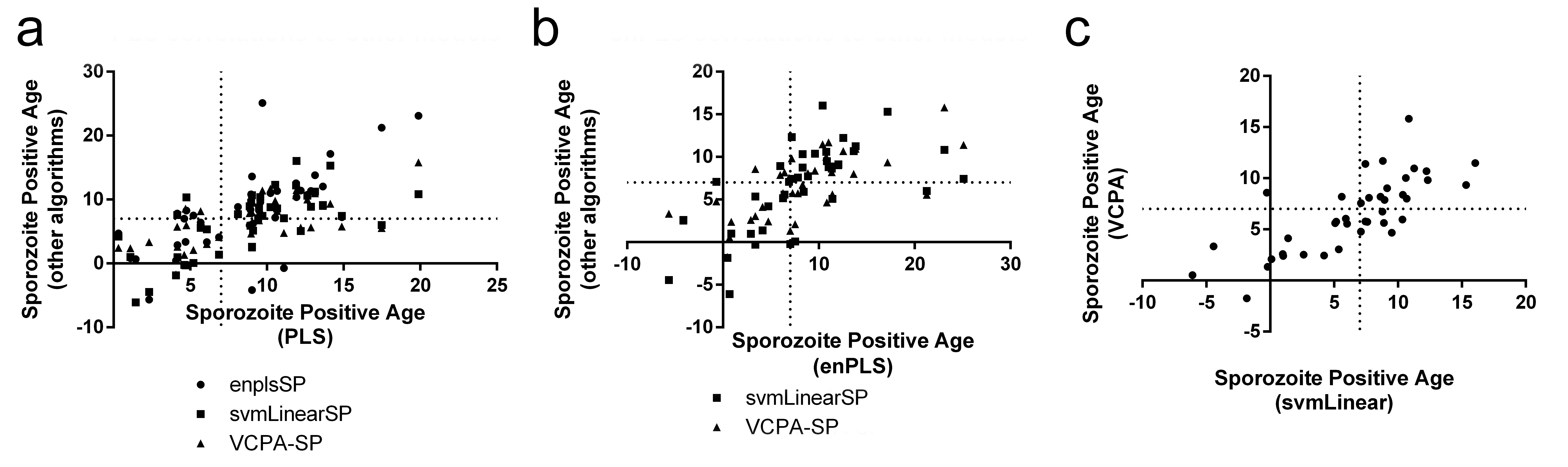

Supplement: Supplementary file 9 — Correlation plot of ages of sporozoite-positive mosquitoes. The predicted ages of expected old, sporozoite-positive mosquitoes for each of the four algorithms are shown. Partial least squares compared to enpls, svmLinear, VCPA (a), enpls compared to svmLinear and VCPA (b), and svmLinear compared to VCPA (c) are shown. All models correlated at P < 0.0001 via Pearson’s r. (TIFF 434 kb) [file 13071_2017_2501_MOESM9_ESM.tif]
